# Targeting Sigma-1 and Sigma-2 Receptors in Neuropathic Pain: Pharmacology, Ligand Development, and Translational Progress

**DOI:** 10.3390/brainsci16040371

**Published:** 2026-03-29

**Authors:** Carlo Reale, Giuliana Costanzo, Lorella Pasquinucci, Carmela Parenti

**Affiliations:** Department of Drug and Health Sciences, University of Catania, 95125 Catania, Italy; carlo.reale@phd.unict.it (C.R.); giuliana.costanzo93@gmail.com (G.C.); carmela.parenti@unict.it (C.P.)

**Keywords:** sigma receptors, neuroinflammation, chronic pain, hyperalgesia, allodynia

## Abstract

**Highlights:**

**What are the main findings?**

- Sigma-1 and sigma-2 receptors act as key intracellular modulators of neuropathic pain, with sigma-1 antagonism specifically attenuating peripheral and central sensitization.
- Sigma-2 receptor/TMEM97 interaction restores proteostatic balance and reduces neuronal vulnerability driven by the integrated stress response (ISR).

**What are the implications of the main findings?**

- Targeting these receptors suppresses neuroinflammation and restores opioid analgesic efficacy, offering a multifaceted approach to pain management.
- The development of clinical candidates like E-52862 (S1RA) demonstrates the feasibility of structure-guided, next-generation therapies for chronic neuropathic pain.

**Abstract:**

Background: Neuropathic pain remains a major unmet clinical challenge. Growing evidence identifies sigma receptors (σRs) as pivotal intracellular modulators of maladaptive stress signaling, positioning them as promising non-opioid targets for chronic pain management. Notably, despite the pleiotropic nature of σRs in regulating diverse cellular pathways—which might theoretically suggest a high risk of off-target effects—current selective antagonists have demonstrated remarkable safety and tolerability profiles. Sigma-1 and sigma-2 receptors (σ1R and σ2R) are molecularly and functionally distinct proteins that regulate neuronal excitability, proteostasis, and neuroimmune communication, all mechanisms that characterize neuronal excitability and cellular stress adaptation. σ1R acts as a ligand-operated molecular chaperone at the mitochondria-associated endoplasmic reticulum membrane. Extensive preclinical data demonstrate that σ1R antagonism attenuates peripheral and central sensitization, suppresses neuroinflammation, and restores opioid analgesic efficacy. These findings are supported by the advanced clinical candidate E-52862, which has shown efficacy and a favorable safety profile in neuropathic pain conditions. σ2R, identified as transmembrane protein 97 (σ2R/TMEM97), functions as a regulator of cholesterol trafficking, lysosomal integrity, and integrated stress response (ISR). σ2R modulation alleviates neuropathic pain by restoring proteostatic balance and reducing ISR-driven neuronal vulnerability rather than directly suppressing excitability. Emerging σ2R ligands such as FEM-1689, UKH-1114, and CM-398 provide compelling proof-of-concept for durable, disease-modifying analgesia. Methods: A structured literature search was conducted using PubMed, Scopus, and Web of Science to identify studies published within the last decade describing σ1R and σ2R/TMEM97 biology, ligand development, and their preclinical or clinical evaluation in neuropathic pain. Reference lists were manually screened to ensure comprehensive coverage. Conclusions: This review synthesizes pharmacology, ligand development, and translational evidence supporting σRs as next-generation targets for neuropathic pain therapy, highlighting convergent roles of σ1R and σ2R in pain chronification and outlining future directions for structure-guided therapeutic strategies.

## 1. Introduction

**Figure 1:**
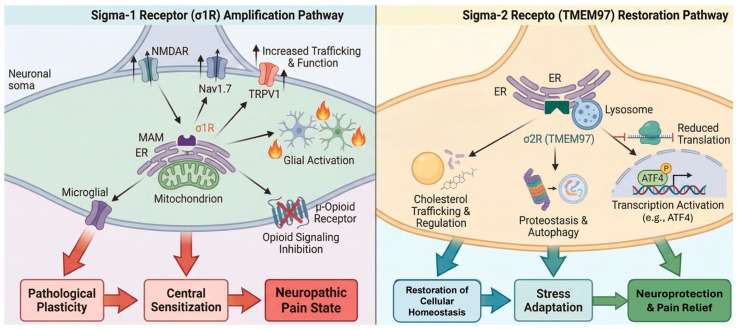
Role of σRs in neuropathic pain: a tale of the two receptors.

Neuropathic pain (NP) remains a clinical challenge, affecting approximately 7–10% of the global population. Unlike nociceptive pain, NP arises from direct injury or dysfunction within the somatosensory nervous system, manifesting through maladaptive phenomena such as hyperalgesia and allodynia [1,2]. Current management relies on a stepwise pharmacological approach. First-line treatments include gabapentinoids, serotonin–norepinephrine reuptake inhibitors, and tricyclic antidepressants, followed by second-line options such as topical agents (lidocaine, capsaicin) and weak opioid-like analgesics (e.g., tramadol, tapentadol). Strong opioids are reserved for severe or refractory cases. Despite these therapeutic strategies, a substantial proportion of patients experience inadequate pain relief or dose-limiting adverse effects, underscoring the need for mechanistically novel targets capable of addressing the biological drivers of pain chronification rather than symptom suppression alone [1,2]. In this context, sigma receptors (σRs) have emerged as promising modulators of pathological pain signaling. Unlike conventional analgesic targets, σRs do not directly suppress nociceptive transmission but instead modulate intracellular stress-adaptive pathways that drive maladaptive plasticity in neuropathic pain [3] (Figure 1).

By the early 1990s, radioligand binding studies defined two pharmacologically distinct σRs subtypes: sigma-1 and sigma-2 receptors (σ1R and σ2R). The nomenclature of σ1R has evolved significantly since its initial description. Originally identified in 1976 by Martin and colleagues, σ1R was initially misclassified as an opioid receptor subtype [4]. However, subsequent work by Su and Tam demonstrated that certain benzomorphans produced effects through naloxone-insensitive pathways, establishing σ1R as a unique receptor population [5]. σ1R exhibited high affinity for [^3^H]-(+)-pentazocine and was cloned in 1996 as a unique ligand-operated chaperone protein with no homology to classical neurotransmitter receptors [6]. In contrast, σ2R, preferentially labeled by [^3^H]-ditolylguanidine, remained molecularly uncharacterized for decades until its identification as transmembrane protein 97 (TMEM97) in 2017 [7]. Collectively, σ1R and σ2R/TMEM97 function as intracellular “stress-gatekeepers”. They regulate calcium homeostasis, proteostasis, and lipid metabolism—processes central to the chronification of neuropathic pain [8]. While underlying molecular pathways are discussed to provide a mechanistic context, the primary focus of this review is to comprehensively outline the recent advancements in pharmacological ligand development. We examine the medicinal chemistry evolution of σR modulators and their efficacy in preclinical and clinical models of neuropathic pain.

## 2. Methods

A structured literature search was conducted using PubMed, Scopus, and Web of Science to identify studies published within the last decade describing σ1R and σ2R/TMEM97 biology, ligand development, and preclinical or clinical evaluation in neuropathic pain. Search terms included combinations of sigma-1 receptor, sigma-2 receptor, TMEM97, sigma ligands, neuropathic pain, analgesia, integrated stress response, and medicinal chemistry. Eligible studies included molecular and structural analyses, medicinal chemistry reports, behavioral pharmacology studies, and clinical trials. Reference lists were manually screened to ensure comprehensive coverage. Extracted data included ligand structure and selectivity, mechanistic pathways, behavioral outcomes, pharmacokinetics, and translational relevance. In this paper, we have used generative artificial intelligence (GenAI, Gemini) to assist with Figure 1 and Figure 6 and GA.

## 3. Sigma-1 Receptor

### 3.1. Distribution and Molecular Architecture

σ1R is a unique, ligand-operated integral membrane protein that bears no structural homology to G-protein-coupled receptors (GPCRs) or ion channels. The crystallization of human σ1R has revealed a trimeric architecture with a single transmembrane domain, providing the structural basis for ligand design [9,10]. The anatomical distribution of σ1R closely mirrors key nodes involved in the development and maintenance of neuropathic pain. Indeed, in the context of pain processing, σ1R is widely distributed across central and peripheral nociceptive circuits. High expression levels have been identified in dorsal root ganglia (DRG)—predominantly in small-to-medium diameter primary sensory neurons (soma and axons) and in satellite glial cells—and in the superficial dorsal horn of the spinal cord (laminae I–II). This distribution underlies both peripheral and central contributions to pathological pain states [11]. Importantly, σ1R expression is dynamic: its levels increase in DRG neurons after inflammatory insults [12] and are upregulated in spinal astrocytes during the development of mechanical allodynia [13,14]. At the subcellular level, σ1R principally localizes to the mitochondria-associated endoplasmic reticulum membrane (MAM), where it functions as a molecular chaperone [15]. Under conditions of agonist binding or cellular stress, σ1R dissociates from BiP (binding immunoglobulin protein) and modulates a variety of client proteins. This chaperone activity regulates ER–mitochondrial Ca^2+^ transfer and calcium homeostasis; in neuropathic pain models, σ1R activation in glia (notably astrocytes) increases intracellular Ca^2+^, promotes release of gliotransmitters and pro-inflammatory cytokines, and contributes to the maintenance of allodynia [16]. Under stress conditions, σ1R can translocate from the MAM to the plasma membrane, where it stabilizes and traffics pro-nociceptive ion channels (e.g., voltage-gated sodium (Nav1.7) and Transient Receptor Potential Vanilloid 1 (TRPV1)) and thereby enhances neuronal excitability and ectopic firing in DRG neurons [17]. In peripheral nerves, σ1R expression rises in Schwann cells after injury, promoting a pro-inflammatory peri-axonal milieu that sustains maladaptive regeneration and ectopic nociceptive input [18]. At supraspinal nuclei (periaqueductal gray (PAG)/rostral ventromedial medulla (RVM)), it acts as a negative regulator of endogenous μ-opioid receptor (MOR) signaling, limiting descending analgesic control [19]. σ1R activity in limbic structures, such as the anterior cingulate cortex (ACC) and amygdala, supports the affective-motivational and fear-related components of pain, and in the nucleus accumbens (NAc) influences dopaminergic signaling linked to motivation and anhedonia in chronic neuropathy. These multi-level actions explain why σ1R antagonists both attenuate peripheral and central sensitization (e.g., by blocking ion-channel trafficking, reducing glial reactivity and cytokine release, suppressing wind-up, and unmasking endogenous opioid analgesia) and mitigate affective and motivational comorbidities of chronic pain [20] (Table 1).

### 3.2. Involvement of Sigma-1 Receptor in Neuropathic Pain Mechanisms

#### 3.2.1. Modulation of Ion Channels and Neurotransmission

Rather than acting as a classical effector receptor, σ1R functions as a context-dependent molecular chaperone that orchestrates the coupling of multiple signaling pathways in response to cellular stress. However, upon agonist binding or cellular stress, σ1R dissociates to modulate a diverse array of “client” proteins. This chaperoning activity is a fundamental driver of neuronal excitability, as σ1R regulates the trafficking and plasma membrane stabilization of several pro-nociceptive ion channels [17].

Among its principal targets are Nav channels and TRP channels. σ1R increases the density of Nav1.7 channels at the plasma membrane, thereby lowering the threshold for action potential firing in injured nociceptors [21]. In addition, σ1R activation has been linked to the modulation of other sodium channel subtypes, such as Nav1.9, enhancing slow sodium currents and increasing action potential firing frequency in small-diameter dorsal root ganglion (DRG) neurons [22].

σ1R also facilitates the activation of TRP channels involved in nociceptive transduction, including Transient Receptor Potential Ankyrin 1 (TRPA1) and Transient Receptor Potential Vanilloid 1 (TRPV1). In particular, σ1R interacts with TRPV1 in a calmodulin-dependent manner, promoting channel activity under pathological conditions such as chemotherapy-induced peripheral neuropathy (CIPN) [23]. Conversely, σ1R antagonism promotes receptor dissociation from TRPV1, enabling calmodulin binding and subsequent channel desensitization, a mechanism that effectively reverses the mechanical hyperalgesia induced by inflammatory mediators [24]. Beyond ion channels, σ1R also modulates excitatory synaptic transmission in the central nervous system. At the postsynaptic level, σ1R promotes phosphorylation and synaptic stabilization of the GluN1 subunit of N-methyl-D-aspartate receptors (NMDARs), thereby amplifying excitatory neurotransmission and contributing to central sensitization [25]. Importantly, these modulatory effects become functionally relevant primarily under pathological conditions, which explains why σ1R antagonism selectively attenuates neuropathic hypersensitivity while largely sparing physiological nociception.

Notably, these modulatory effects become functionally relevant mainly under pathological conditions, thereby explaining why σ1R antagonism selectively attenuates neuropathic hypersensitivity while sparing physiological nociception.

#### 3.2.2. Peripheral Sensitization: DRG-Level Modulation

σ1R acts as a critical modulator of nociceptive signaling within the peripheral nervous system, particularly at the level of DRG. σ1R is densely expressed in the somata of primary sensory neurons (PSNs), including both peptidergic and non-peptidergic nociceptors, as well as in satellite glial cells [18]. Under pathological conditions, such as peripheral nerve injury, σ1R expression is upregulated or translocated to the plasma membrane, contributing to the development of neuropathic pain and neuronal hyperexcitability [26]. At this level, σ1R influences peripheral sensitization through the coordinated modulation of nociceptive ion channels described above, including Nav and TRP family members, thereby enhancing excitability in DRG neurons. In addition to these direct neuronal effects, σ1R also regulates neuroimmune communication within the DRG. Specifically, it modulates the expression of chemokines such as C-C motif chemokine ligand 2 (CCL2), promoting the recruitment and infiltration of macrophages and CD4^+^ T cells and sustaining the neuroinflammatory milieu associated with chronic pain states [26].

Conversely, pharmacological blockade of σ1R or genetic silencing of the receptor in PSNs normalizes neuronal hyperexcitability and significantly attenuates pain behaviors without altering baseline sensory thresholds [17,27].

#### 3.2.3. Central Sensitization: NMDA Receptor Potentiation

σ1R plays a pivotal role in the induction and maintenance of central sensitization by modulating glutamatergic neurotransmission, specifically through the potentiation of NMDARs in the spinal dorsal horn [28]. Upon activation during cellular stress or nociceptive signaling, σ1Rs translocate from the endoplasmic reticulum to the plasma membrane, where they physically interact with the GluN1 subunit of the NMDAR [29]. This interaction facilitates the phosphorylation of GluN1 (pGluN1) at specific protein kinase C (PKC) and protein kinase A (PKA)-dependent sites, thereby enhancing NMDAR calcium permeability and trafficking to the synaptic membrane [30,31]. Furthermore, σ1R activation inhibits small-conductance calcium-activated potassium (SK) channels, which normally act to shunt NMDAR-mediated currents; the removal of this inhibitory brake amplifies calcium influx and promotes long-term potentiation (LTP) and spinal wind-up [32]. Additionally, σ1Rs regulate the coupling of NMDARs with cannabinoid CB1 receptors (CB1Rs) and histidine triad nucleotide-binding protein 1 (HINT1), acting as a “safety switch” that prevents cannabinoid-mediated inhibition of NMDAR activity, thus sustaining excitatory signaling [33]. Collectively, these molecular mechanisms lead to a state of spinal neuronal hyperexcitability and persistent pain hypersensitivity [34].

### 3.3. Modulation of Opioid Analgesia

σ1R has been identified as a tonically active anti-opioid system that physically associates with MOR to negatively regulate its signaling efficacy. Pharmacological blockade of the σ1R disrupts this inhibitory interaction, thereby potentiating opioid-induced antinociception without increasing MOR binding affinity, a mechanism that essentially “releases the brake” on opioid signaling [35,36]. Specifically, this crosstalk is modulated by a multiprotein complex involving the Histidine Triad Nucleotide-binding Protein 1 (HINT1). Upon MOR activation, nitric oxide production triggers a redox zinc switch in the RGSZ2 protein, which recruits the redox sensor PKCγ to HINT1. This cascade enables the interaction of the σ1R—which is bound to the NMDAR NR1 subunit—with the MOR-HINT1 complex, thereby restraining opioid signaling [37]. This modulation is mechanistically underpinned by the ability of σ1R antagonists to transiently disrupt the σ1R-NR1 association, facilitating the binding of negative regulators like Ca^2+^-calmodulin to the NMDAR [38,39]. This molecular disinhibition effectively uncouples MOR from the negative feedback exerted by NMDARs, which is particularly relevant for mitigating the development of analgesic tolerance and restoring morphine efficacy in tolerant states [40]. Pharmacological inhibition of σ1R has been shown to restore opioid sensitivity and delay the development of analgesic tolerance. This synergy has led to the development of bifunctional ligands (MOR agonist/σ1R antagonist), which achieve potent analgesia at lower doses, effectively widening the therapeutic window by reducing the risk of respiratory depression and opioid-induced hyperalgesia [41].

### 3.4. Sigma-1 Receptor Ligands

The development of σ1R ligands has progressed from early pharmacological probes to highly selective, clinically oriented antagonists. Antagonism of σ1R reduces spinal sensitization, attenuates microglial/astrocytic activation and macrophage infiltration into injured DRG, and thereby decreases mechanical allodynia and thermal hyperalgesia across multiple neuropathic paradigms (nerve injury-, chemotherapy-, and diabetic-induced neuropathy) [42]. Conversely, selective σ1R agonists can stabilize receptor conformations that enhance chaperone activity and, in some models, show neuroprotective effects but may enhance nociceptive signaling in sensitized pathways. This functional dichotomy underpins the field’s emphasis on antagonists as analgesics and on careful functional profiling (agonist/antagonist) during lead optimization [43,44,45].

#### 3.4.1. Foundational Probes and the Agonist-Antagonist Paradigm

**Figure 2:**
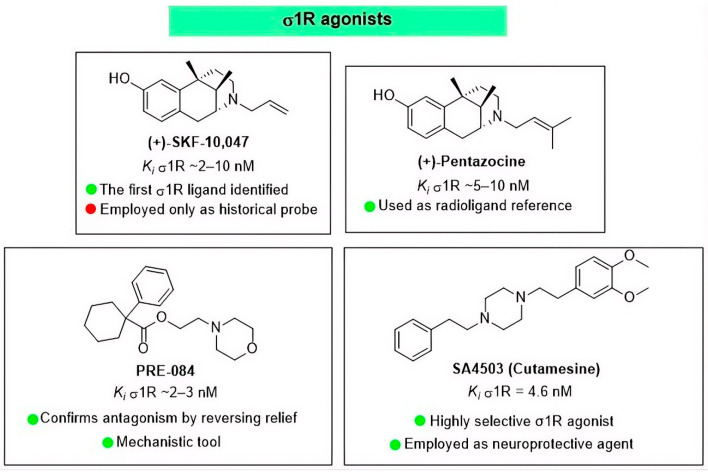
σ1R agonists.

The initial pharmacological characterization of the σ1R relied on classical benzomorphan agonists such as (+)-SKF-10,047 (N-allylnormetazocine) and (+)-pentazocine [10,46]. While foundational for defining the receptor’s existence, these molecules possess significant liabilities, including psychotomimetic effects and cross-reactivity with opioid receptors [47,48]. To overcome these off-target limitations, structurally distinct and highly selective σ1R agonists, most notably SA4503 (cutamesine), were subsequently developed [49]. This high selectivity has not only facilitated its use as a radioligand ([^11^C]SA4503) for in vivo positron emission tomography (PET) mapping of central σ1 receptors [50] but has also highlighted its broad therapeutic utility, demonstrating robust neuroprotective, antidepressant-like, and cognitive-enhancing properties across various CNS disease models [51]. In neuropathic pain models, these molecules serve as critical “pharmacological mirrors”: while they may not induce spontaneous nociception in naïve phenotypes, they effectively reverse the anti-allodynic and anti-hyperalgesic effects of selective σ1R antagonists, thereby confirming that the observed analgesia is specifically mediated by σ1R blockade [52] (Figure 2).

#### 3.4.2. Classical Antagonists and Analytical Ligands

**Figure 3:**
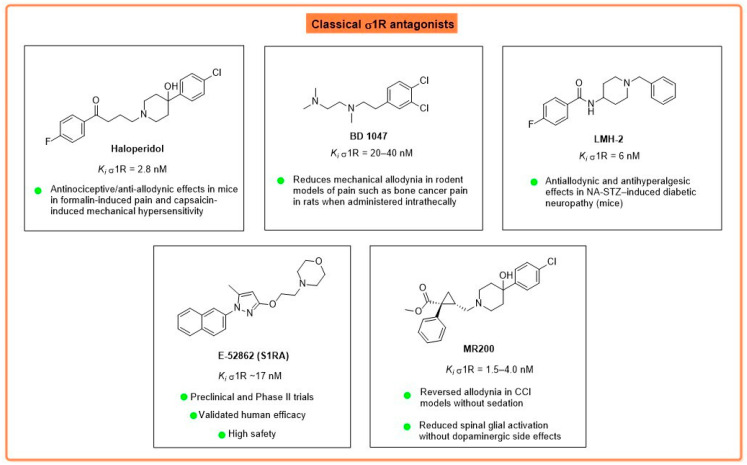
Classical σ1R antagonists.

Haloperidol (HAL), a classical neuroleptic butyrophenone, acts as a non-selective antagonist with high nanomolar affinity for both σ1R (K*_i_* = 2–6 nM) and dopamine D2 receptors [53]. While in vivo studies utilizing the Chronic Constriction Injury (CCI) of the sciatic nerve and the streptozotocin-induced diabetic neuropathy (STZ-DPN) models have demonstrated that HAL significantly reduces mechanical allodynia and hyperalgesia [54], its therapeutic utility is severely limited by extrapyramidal side effects (catalepsy and motor imbalance) resulting from striatal D2 receptor blockade [53] (Figure 3).

To overcome this off-target profile, BD 1047 was developed as a selective σ1R antagonist (K*_i_* = 0.9 nM) with negligible affinity for dopaminergic or opioid receptors [55]. In vitro and in vivo studies indicate that BD 1047 exerts antinociceptive effects by inhibiting σ1R-mediated NMDAR potentiation [55]. In a rat model of bone cancer pain induced by intramedullary injection of Walker 256 carcinoma cells, intrathecal administration of BD 1047 significantly reduced mechanical allodynia and decreased spinal microglial activation, as indicated by diminished ionized calcium binding adaptor molecule 1 (Iba-1) expression, by lowering p38 mitogen-activated protein kinases (MAPK) phosphorylation, and tumor necrosis factor-alpha (TNF-α) levels [56]. It has demonstrated robust efficacy in reducing mechanical allodynia also in other NP models, including spinal nerve ligation (SNL) and chronic compression of the dorsal root ganglion (CCD), and in capsaicin-induced headache models [55,57] (Figure 3).

To combine the antinociceptive potency of HAL with the safety profile of selective σ1R antagonist BD 1047, LMH-2 (1-benzylpiperidin-4-yl)-4-fluorobenzamide) was developed as a novel HAL analog [53]. In vitro binding and in silico docking studies confirm that LMH-2 retains high affinity for σ1R (K*_i_* = 6.0 nM), interacting with Glu172 and Tyr103 residues, but exhibits a significantly lower affinity for D2 receptors (K*_i_* = 202 nM; 58-fold lower than HAL) [53]. In a mouse model of diabetic neuropathy induced by nicotinamide and streptozotocin (NA-STZ), LMH-2 produced dose-dependent antiallodynic and antihyperalgesic effects superior to those of gabapentin and HAL, without inducing catalepsy or altering locomotor activity [53]. Recent behavioral and molecular docking studies suggest that LMH-2 may also interact with the TRPV1 channel (effects blocked by capsazepine) and modulate the opioid/NMDAR complex, providing a multi-target mechanism for controlling diabetic neuropathic pain without the adverse effects associated with classical neuroleptics [57] (Figure 3).

(+)-MR200, chemically identified as (+)-methyl (1*R*,2*S*)-2-{[4-(4-chlorophenyl)-4-hydroxypiperidin-1-yl]methyl}-1-phenylcyclopropanecarboxylate, is a highly potent and selective σ1R antagonist (K*_i_* values 1.5–4.0 nM) that distinguishes itself from its parent compound HAL by lacking dopaminergic affinity [58,59]. Pharmacologically, the compound does not show antinociceptive effects in acute thermal thresholds when administered alone but potentiates significant opioid analgesia and exhibits therapeutic efficacy in sensitizing pain states [60]. In vivo studies have established (+)-MR200’s ability to dose-dependently reverse mechanical allodynia, thermal hyperalgesia, and edema in carrageenan-induced inflammatory models [60]. Furthermore, in the CCI model of neuropathic pain, (+)-MR200 exerts robust antiallodynic and neuroprotective effects by modulating spinal glial activity; specifically, it dampens the reactive gliosis of astrocytes and microglia and normalizes the expression of Connexin 43 (Cx43), therefore inhibiting the pathological upregulation of gap junction-mediated intercellular communication that contributes to the maintenance of chronic pain [61] (Figure 3).

E-52862 (also known as S1RA or MR309) exhibits high affinity for human σ1R (K*_i_* = 17 nM) and exceptional selectivity, with a σ1R/σ2R binding ratio greater than 550 and negligible affinity for a panel of over 170 other receptors, transporters, enzymes, and ion channels [62]. In vitro and ex vivo studies have shown that E-52862 inhibits the spinal wind-up phenomenon—a measure of spinal cord hyperexcitability—induced by repetitive nociceptive stimulation [35]. At the molecular level, this antinociceptive activity is associated with the prevention of NMDAR GluN2B subunit (specifically at Tyr1472 and Ser1303) phosphorylation and the downregulation of extracellular signal-regulated kinase (ERK1/2) phosphorylation in the dorsal horn of the spinal cord [63]. Furthermore, E-52862 reduces the spinal expression of pro-inflammatory cytokines such as TNF-α and IL-1β following nerve injury [64]. Additionally, E-52862 has been shown to potentiate opioid antinociception (e.g., morphine) without exacerbating opioid-related side effects, likely by modulating the σ1R-MOR interaction [64]. Recent patch-clamp studies in DRG neurons also suggest that E-52862 can modulate excitability by inhibiting fast-inactivating sodium currents while enhancing slow-inactivating currents mediated by Nav1.9 [25]. In vivo, E-52862 has demonstrated robust, dose-dependent antiallodynic and antihyperalgesic effects across a broad spectrum of neuropathic pain models, without altering basal nociceptive thresholds [63]. Efficacy has been consistently observed in models of peripheral nerve injury, including partial sciatic nerve ligation (PSNL), CCI, and spared nerve injury (SNI) [52]; in chemotherapy-induced peripheral neuropathy models triggered by paclitaxel, oxaliplatin, and cisplatin, where it prevents mechanical and cold allodynia and limits mitochondrial dysfunction; and in STZ-DPN and in central neuropathic pain following spinal cord injury, where repeated administration during the acute phase prevented the development of pain behaviors for up to 28 days post-injury [64] (Figure 3, Table 2).

#### 3.4.3. Advancement in Sigma 1 Receptor Antagonists (2015–2026)

While classical antagonists provided foundational proof-of-concept for σ1R modulation in neuropathic pain, their clinical translation was often hindered by off-target liabilities (e.g., D2-mediated extrapyramidal effects) or suboptimal pharmacokinetics. The emerging antagonists developed over the last decade (2015–2026) represent a rational evolution aimed at overcoming these specific barriers. These next-generation compounds advance beyond classical ligands by prioritizing enhanced blood–brain barrier (BBB) penetration, superior metabolic stability, and refined selectivity profiles designed to mitigate specific safety liabilities, such as hERG-related cardiotoxicity. The following section details these emerging candidates, highlighting their structural improvements and pharmacological advantages.

**Figure 4:**
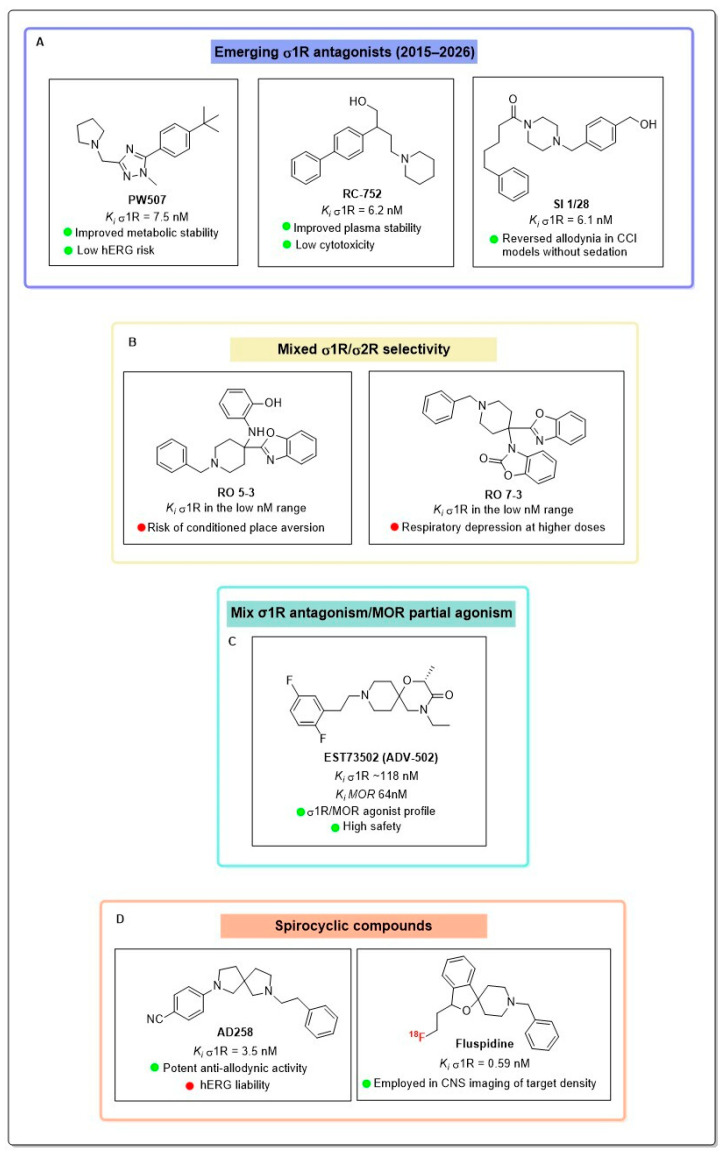
Emerging antagonists with optimized pharmacokinetics: panel (**A**). Mixed σ1R/σ2R and σ1R/MOR bifunctional ligands: panels (**B**,**C**). Spirocyclic compounds: panel (**D**).

PW507 (TNX-4900) is a highly potent and selective small-molecule σ1R antagonist (K*_i_* = 7.5 nM) with greater than 100-fold selectivity over σ2R and low affinity for the human Ether-à-go-go-Related Gene (hERG) channel involved in cardiotoxicity risks [65]. Structurally based on a trisubstituted 1,2,4-triazole scaffold, PW507 binds to the σ1R orthosteric site via a critical salt bridge with Glu172, effectively blocking σ1R-mediated sensitization [66]. In vitro, it demonstrates high metabolic stability in human liver microsomes and substantial blood–brain barrier permeability (brain/plasma ratio ~12–15) [65]. The antinociceptive properties of PW507 have been primarily established using the STZ-induced diabetic neuropathy model, where both acute and chronic intraperitoneal (i.p.) administration significantly attenuated mechanical allodynia and thermal hyperalgesia without inducing tolerance or observable toxicity. Additionally, efficacy data from previous proof-of-concept studies indicate that PW507 is also effective in mitigating paclitaxel-induced neuropathic pain and formalin-induced inflammatory pain [65] (Figure 4).

RC-752 is a small molecule belonging to the class of 2-aryl-4-aminobutanol derivatives, identified as a potent and selective σ1R antagonist [66]. Functionally, RC-752 has been identified as a dual modulator of σ1R and aquaporins (AQPs), capable of influencing AQP-mediated water and hydrogen peroxide permeability in HeLa cells under oxidative stress conditions, suggesting a potential role in mitigating oxidative damage associated with neuroinflammation [66,67]. The compound displayed high metabolic stability in mouse plasma and demonstrated no cytotoxicity in normal human cell lines or in an in vivo zebrafish model, indicating a favorable safety window for therapeutic application [66]. Pharmacokinetic evaluations in silico suggest that RC-752 possesses favorable BBB permeability, achieving high concentrations in the central nervous system (CNS), which is essential for its central antinociceptive activity. RC-752 has shown robust analgesic efficacy in rodent models of persistent and neuropathic pain. In the mouse formalin test, i.p. administration induced dose-dependent antinociception in both Phase I and Phase II of the test, with significantly higher potency during the inflammatory phase, indicating an effect on central sensitization [66]. In the SNL model, RC-752 reversed mechanical allodynia, restoring paw withdrawal thresholds to pre-injury levels without altering basal nociception [66]. The rapid onset and sustained duration of action further support its potential as a non-opioid modulator of neuropathic pain (Figure 4).

SI 1/28 [1-(4-{[4-(hydroxymethyl)phenyl]methyl}piperazin-1-yl)-5-phenylpentan-1-one oxalate] is a novel benzylpiperazine derivative developed as a highly selective σ1R antagonist. In vitro radioligand binding studies determined that SI 1/28 possesses high nanomolar affinity for σ1R (K*_i_* = 6.1 nM) and a selectivity profile over σ2R (K*_i_* = 2583 nM), with a selectivity ratio of approximately 423-fold. Pharmacologically, SI 1/28 modulates nociceptive signaling by inhibiting σ1R-mediated sensitization, a key mechanism in the maintenance of chronic pain states [68,69]. In vivo efficacy was evaluated using multiple murine models of nociception. In the CCI model of neuropathic pain, i.p. administration of SI 1/28 produced dose-dependent anti-allodynic effects, demonstrating efficacy comparable to gabapentin at higher doses. Additionally, the compound displayed significant antinociceptive activity in models of inflammatory pain, specifically reducing nociceptive behaviors in Phase II of the formalin test [68,69]. Notably, unlike traditional analgesics such as opioids or gabapentinoids, SI 1/28 exhibited a favorable safety profile in behavioral liability profiling: it showed no significant effects on spontaneous locomotion, motor coordination (rotarod assay), or respiratory rate, and lacked reinforcing properties in the conditioned place preference (CPP) assay, suggesting a low potential for abuse or sedation [68,69] (Figure 4).

RO-5-3 and RO-7-3 are synthesized via a three-component Ugi reaction and designed through a scaffold hopping strategy based on the selective σ1R ligand UVM-147 [70]. In vitro radioligand competition binding assays demonstrated that both analogs possess nanomolar affinity for σ1R (K*_i_* = 27 nM for RO-5-3 and 24 nM for RO-7-3), comparable to the parent compound, though they exhibit reduced selectivity over the σ2R (selectivity indices of approximately 9- and 15-fold, respectively). Despite this reduced subtype selectivity, both compounds displayed a favorable off-target profile against a broad panel of CNS receptors [70]. In vivo efficacy was evaluated using the CCI model of neuropathic pain and the formalin assay in mice. In the CCI model, subcutaneous administration of RO-5-3 produced a significant, dose-dependent attenuation of mechanical allodynia. Conversely, RO-7-3 showed anti-allodynic potential, but it lacked significant efficacy at higher doses, potentially due to confounding motor effects. Both ligands effectively reduced nociceptive behaviors in Phase II of the formalin assay, consistent with the role of σ1R antagonism in modulating central sensitization [70]. Regarding safety and mechanism of action, behavioral assays revealed distinct profiles for the two analogs. RO-5-3 induced mild respiratory depression without locomotor impairment but elicited conditioned place aversion (CPA), a side effect potentially linked to its higher relative engagement of σ2R signaling pathways. In contrast, RO-7-3 caused transient respiratory depression and locomotor impairment but did not induce CPA. These findings highlight the critical importance of σ1R/σ2R selectivity in the development of non-opioid analgesics for neuropathic pain [70] (Figure 4).

EST73502 (also known as WLB-73502 or ADV-502) represents a first-in-class, bispecific small molecule designed to address the limitations of traditional opioid therapy through a dual mechanism of action: partial agonism at MOR and antagonism at σ1R [71]. In vitro binding assays demonstrate that the compound possesses balanced nanomolar affinity for both human recombinant targets (K*_i_* values of 64 nM for MOR and 118 nM for σ1R) while maintaining high selectivity against a broad panel of off-targets. Functionally, EST73502 exhibits G-protein-biased signaling at MOR: it acts as a partial agonist in the G-protein pathway (cAMP inhibition) with low intrinsic efficacy but negligible or undetectable β-arrestin-2 recruitment, a pathway traditionally associated with opioid-related adverse events [71]. This signaling profile sharply contrasts with classical full opioid agonists like morphine and oxycodone, which robustly recruit the β-arrestin pathway [72]. The low intrinsic efficacy for β-arrestin recruitment, combined with partial G-protein activation, is believed to critically contribute to the minimization of typical opioid-related adverse effects [71]. In vivo studies have validated this multimodal approach, demonstrating robust dose-dependent antinociception in various rodent models. Specifically, in models of neuropathic pain, such as PSNL and SNI, EST73502 exhibited strong efficacy, attenuating mechanical allodynia with a potency superior to morphine and comparable to oxycodone [73]. The synergistic contribution of both targets to the observed analgesia was confirmed by reversal studies, where the antinociceptive effects were blocked by both the MOR antagonist naloxone and the selective σ1R agonist PRE-084 [3]. Furthermore, pharmacological evaluations highlight a significantly improved safety profile compared to strong opioids; repeated administration of EST73502, indeed, in chronic pain models showed no development of analgesic tolerance [71]. The σ1R antagonistic component provides a crucial secondary layer of regulation through its modulation of NMDAR coupling, which is highly relevant to this lack of opioid tolerance. Repeated opioid treatment typically activates pain facilitatory pathways through specific phosphorylation of NMDAR, which in turn stimulates kinase cascades that phosphorylate MOR and disrupt its coupling to G-proteins [71]. σ1R physically binds to the NR1 subunit of the NMDAR, and its blockade by a σ1R antagonist diminishes this affinity, allowing the negative regulator calcium-calmodulin to bind and inhibit the NMDAR, thereby blocking the detrimental NMDAR-to-MOR signaling. Additionally, the compound displayed a reduced propensity for physical dependence (measured by naloxone-precipitated withdrawal), markedly lower inhibition of gastrointestinal transit, and a lack of respiratory depression or proemetic effects at equianalgesic doses [71] (Figure 4).

AD258 represents a prominent lead within a series of 2,7-diazaspiro [4.4]nonane derivatives designed as σR ligands. Pharmacologically, AD258 acts as a mixed, high-affinity ligand for both σR subtypes, with low nanomolar binding affinity for σ1R (K*_i_* = 3.5 nM) and σ2R (K*_i_* = 2.6 nM) [74]. Selectivity profiling confirmed that AD258 does not exhibit significant affinity for other pain-related targets, such as opioid, cannabinoid, serotonergic, or NMDA receptors. Mechanistic studies, employing the phenytoin shift assay, indicated that AD258 functions as a σ1R antagonist, a profile further corroborated by in vivo functional experiments where the σ1R agonist PRE-084 fully reversed the compound’s antiallodynic effects [74]. In in vitro safety assessments, AD258 showed negligible cytotoxicity in human corneal epithelial (HCE) cells at pharmacologically relevant concentrations, although reduced cell viability was observed at high concentrations (100 µM). Metabolic stability assays in human and mouse liver microsomes revealed a moderate intrinsic clearance profile (CL_int_ > 50 and 150 µL/min/mg protein). However, the compound exhibited high inhibition of the hERG potassium channel (IC_50_ = 0.085 µM), highlighting a potential liability for QT prolongation [74]. The in vivo analgesic efficacy of AD258 was characterized using a mouse model of capsaicin-induced mechanical allodynia, a standard paradigm for central sensitization. In this model, AD258 demonstrated high potency, achieving a full reversal of mechanical hypersensitivity at low doses, significantly outperforming the reference σ1R antagonist BD-1063. Furthermore, at an analgesic dose, AD258 did not induce motor coordination deficits in the rotarod test, confirming that its antinociceptive activity is not confounded by sedation or motor impairment. These findings position AD258 as a potent mixed σ1R/σ2R ligand potentially useful for the management of neuropathic pain states derived from central sensitization [74] (Figure 4).

Fluspidine (1′-benzyl-3-(2-fluoroethyl)-3H-spiro[benzofuran-1,4′-piperidine]) has been developed as a radioligand for the selective positron emission tomography (PET) imaging of σ1R. Pharmacologically, it exhibits sub-nanomolar affinity (K*_i_* = 0.59 nM) and exceptional selectivity for the σ1R over the σ2R and other central nervous system targets. Extensive in vitro and in vivo studies in mice, piglets, and non-human primates have confirmed its metabolic stability in the brain and high specific uptake in σ1R-rich regions [75] (Figure 4).

### 3.5. Clinical Candidates: From Bench to Bedside

**Figure 5:**
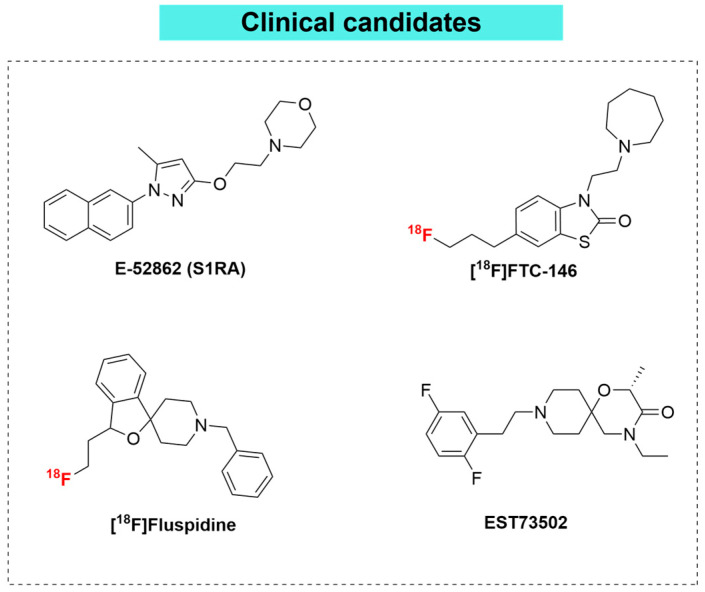
Clinical candidates.

σ1R antagonism has emerged as a validated, non-opioid therapeutic strategy specifically targeting the pathophysiology of neuropathic pain. In this context, E-52862 is a first-in-class, selective σ1R antagonist that has demonstrated a favorable safety and tolerability profile in Phase I clinical trials, exhibiting pharmacokinetics compatible with once-daily oral administration [63]. In Phase II proof-of-concept studies, the molecule has shown potential utility in managing neuropathic pain of various etiologies, particularly in oxaliplatin-induced peripheral neuropathy (OIN), where it significantly reduced acute cold allodynia and the incidence of severe chronic neuropathy (NCI-CTCAE grade ≥ 3, *National Cancer Institute—Common Terminology Criteria for Adverse Events*), while allowing for higher cumulative doses of chemotherapy [76]. Furthermore, in patients with chronic postsurgical pain (CPSP), E-52862 demonstrated superior analgesic efficacy compared to placebo; in painful diabetic neuropathy (PDN) trials, however, it did not achieve statistical significance vs. placebo response, despite an observed reduction in pain intensity. Due to these efficacy hurdles and the suboptimal pharmacokinetic profile (e.g., low oral bioavailability), the clinical development of E-52862 for neuropathic pain has been largely discontinued. However, its clinical journey provided crucial proof-of-concept that paved the way for next-generation analogs. The drug is generally well-tolerated, with the most frequently reported treatment-emergent adverse events being mild-to-moderate dizziness, headache, and nausea, and no significant cardiac toxicity has been reported [77]. Mechanistically, σ1R antagonists attenuate the receptor-mediated amplification of nociceptive signaling—including the modulation of NMDA receptor activity—triggered by peripheral nerve injury [52,78]. Furthermore, the high-affinity antagonist [^18^F]FTC-146 remains in active clinical development, currently undergoing Phase I trials as a diagnostic PET tracer to visualize σ1R upregulation at sites of nerve damage in conditions such as sciatica and complex regional pain syndrome (CRPS), potentially serving as a biomarker for localized pain generators [79,80]. Another clinically relevant radioligand, (*S*)-[^18^F]fluspidine, has been successfully employed to map σ1R density alterations in major depressive disorder and to quantify the receptor occupancy of the therapeutic agonist pridopidine in Huntington’s disease. Within the field of pain research, (*S*)-[^18^F]fluspidine has specifically demonstrated utility in imaging of pain-associated σ1R upregulation in rat models of postoperative pain (partial liver resection), highlighting its sensitivity in detecting peripheral receptor expression changes under analgesic conditions [75]. EST73502 represents a novel, first-in-class bifunctional agent designed to address the complex pathophysiology of neuropathic pain by combining high-affinity σ1R antagonism with partial MOR agonism. This dual mechanism exploits the ability of σ1R blockade to potentiate opioid analgesia, thereby allowing for an opioid-sparing effect that maintains efficacy while mitigating classic opioid-related adverse events [3]. In Phase I clinical trials involving healthy volunteers, EST73502 demonstrated a favorable safety and tolerability profile, exhibiting linear pharmacokinetics and central target engagement (confirmed via pupillometry) without causing serious adverse events or clinically significant QTc prolongation. Unlike first-generation antagonists, EST73502 remains in active clinical development for the management of complex conditions such as neuropathic cancer pain and osteoarthritis, as well as a potential intervention for opioid use disorder. EST73502 illustrates the translational potential of σ1R antagonism as a strategy to enhance the therapeutic index of centrally acting analgesics [44,81] (Figure 5).

### 3.6. Pharmacokinetics and Safety of Sigma-1 Receptor Antagonists

The clinical translation of σ1R antagonists for neuropathic pain critically depends on their pharmacokinetic (PK) properties, safety profile, and the ability to demonstrate target engagement in vivo. Early-stage development of ligands employed UPLC–MS/MS (ultra-performance liquid chromatography—tandem mass spectrometry) analyses to confirm blood–brain barrier (BBB) permeability to establish central exposure as a prerequisite for therapeutic evaluation [82,83]. Similarly, E-52862 was shown to achieve substantial central σ1R occupancy, which correlated with analgesic efficacy in preclinical and early clinical studies [84]. To further address the translational gap between anatomical nerve injury and molecular target engagement, PET radioligands have been developed, most notably [^18^F]FTC-146. This highly selective tracer enables non-invasive visualization of σ1R upregulation associated with nerve injury and neuroinflammation [85,86,87]. Importantly, radiotracer uptake has been shown to correlate with the severity of mechanical allodynia in preclinical and translational models, suggesting that PET imaging may serve as a biomarker for identifying peripheral pain generators in complex clinical conditions such as sciatica and complex regional pain syndrome (CRPS), although broader clinical validation is still required [87]. From a safety perspective, selective σ1R antagonists exhibit a favorable profile compared with conventional analgesics. Unlike opioids or gabapentinoids, compounds such as E-52862 do not induce sedation, motor impairment, respiratory depression, or physical dependence in clinical and preclinical assessments [88,89]. Nevertheless, despite these advantages, E-52862 has encountered limitations in late-stage clinical development. While efficacy was observed in chronic postsurgical pain [77], outcomes in painful PDN were confounded by high placebo responses, complicating efficacy assessment. In addition, E-52862 displays a suboptimal PK profile, characterized by low oral bioavailability (~15%) and the need for relatively high daily doses to maintain approximately 70% receptor occupancy, necessary for a robust analgesic effect [90]. These challenges are not unique to E-52862. Rapid systemic clearance observed for other σ1R ligands, such as CM-304, has further underscored the need for next-generation compounds with improved PK characteristics. In response, newer candidates, including PW507, have been developed, offering enhanced oral bioavailability and improved BBB penetration while preserving σ1R selectivity [84,90]. Interindividual variability also contributes to heterogeneous therapeutic outcomes. Genetic polymorphisms in the sigma non-opioid intracellular receptor 1 (SIGMAR1) gene have been associated with altered somatosensory phenotypes, potentially influencing patient responsiveness to σ1R targeted therapies [91]. Moreover, while clinical efficacy is more consistently demonstrated in peripheral neuropathic pain, the extension of σ1R antagonism to central pain syndromes remains less established. Nonetheless, encouraging preclinical data from spinal cord injury models suggest that central mechanisms may still be amenable to modulation via σ1R blockade [92,93]. To overcome these pharmacological and clinical limitations, σ1R antagonists are increasingly investigated as components of combination therapies. σ1R blockade has been shown to synergistically enhance the antinociceptive efficacy of MOR agonists while attenuating the development of tolerance and dependence [94,95]. Beyond opioids, supra-additive analgesic effects have also been reported when σ1R antagonists are combined with gabapentinoids, consistent with a role in dampening central sensitization and glial reactivity [79]. These findings have motivated the rational design of multi-target-directed ligands (MTDLs) and bifunctional compounds. Notable examples include EST73502 (WLB-73502), which combines partial MOR agonism with σ1R antagonism to achieve potent analgesia with an improved safety profile [71,96], as well as emerging hybrids targeting σ1R alongside histamine H3 receptors [97].

Collectively, these strategies aim to preserve the mechanistic advantages of σ1R modulation while mitigating the PK and dose-related liabilities that have limited the clinical success of first-generation agents.

## 4. Sigma-2 Receptor

### 4.1. Distribution and Molecular Architecture

σ2R, molecularly established as TMEM97 (σ2R/TMEM97), has recently emerged as a key intracellular regulator with substantial therapeutic potential in neuropathic pain. For decades, σ2R was defined solely by its radioligand binding properties, lacking molecular identity or clearly assigned physiological roles. The discovery that σ2R corresponds to TMEM97 has redefined research paradigms by linking the receptor to cholesterol homeostasis, lysosomal integrity, and proteostatic regulation [98]. This shift from a hypothetical binding site to a well-characterized protein enabled rational ligand design and provided a mechanistic rationale for targeting σ2R/TMEM97 in chronic pain conditions marked by metabolic stress and neuronal vulnerability [99]. σ2R/TMEM97 does not couple to classical second-messenger cascades and therefore cannot be interpreted through the classical agonist–antagonist pharmacology [100]. σ2R/TMEM97 ligands act as functional modulators of intracellular stress pathways, particularly the ISR. Under neuropathic conditions, persistent ISR activation elevates p-eIF2α, induces ATF4-driven transcription, and destabilizes proteostasis within DRG neurons [99,101]. σ2R/TMEM97 modulation attenuates these processes, restoring homeostatic signaling and reducing nociceptor sensitization.

Recent transcriptomic, proteomic, and histological studies have delineated the anatomical localization of σ2R/TMEM97 within nociceptive circuits. The receptor is highly concentrated in small-to-medium DRG neurons, where it shows heightened susceptibility to metabolic and proteostatic stress [102,103]. σ2R/TMEM97 expression in satellite glial cells and Schwann cells further implicates σ2R in sensory neuron–glia metabolic coupling and axonal maintenance [98]. Within the spinal cord, σ2R/TMEM97 is strongly expressed in laminae I–II of the dorsal horn, the principal termination zones for nociceptive afferents. This localization suggests a functional role for σ2R/TMEM97 in modulating synaptic integration, central sensitization, and the metabolic stability of dorsal horn interneurons, including inhibitory subpopulations known to be compromised in neuropathic states [95]. At supraspinal levels, σ2R/TMEM97 expression spans multiple pain-processing structures, including the thalamus, PAG, ACC, amygdala, and insula [103]. These regions govern sensory-discriminative, affective-emotional, and descending modulatory components of pain. Given that maladaptive ISR signaling influences affective and cognitive responses to chronic pain [104], σ2R/TMEM97 expression in limbic circuits provides a mechanistic foundation for the ability of σ2R/TMEM97 modulators to alleviate both sensory and affective dimensions of neuropathic pain. The anatomical distribution of σ2R/TMEM97 across peripheral, spinal, and supraspinal circuits is summarized in Table 3.

### 4.2. Sigma 2 Receptor as a Regulator of Proteostasis and the Integrated Stress Response

σ2R/TMEM97 participates in the regulation of ER stress, cholesterol transport, and lysosomal biology, domains intimately linked to neuronal viability and sensory neuron performance [105]. At the molecular level, σ2R/TMEM97 forms a functional trimeric complex with the progesterone receptor membrane component 1 (PGRMC1) and the low-density lipoprotein receptor (LDLR) [106,107]. This macromolecular complex is essential for the rapid cellular internalization of lipoproteins, including LDL and apolipoprotein E (ApoE), as well as toxic protein aggregates like amyloid-beta (Aβ42) monomers and oligomers [108]. Upon internalization into the endo-lysosomal system, σ2R/TMEM97 interacts with the Niemann-Pick C1 (NPC1) protein to regulate the efflux of cholesterol out of lysosomes, thus maintaining intracellular lipid homeostasis. Disruptions in this tightly regulated lipid trafficking can lead to lysosomal lipid storage, lipid raft remodeling, and subsequent induction of ER stress and intracellular calcium (Ca^2+^) dysregulation [106,107].

**Figure 6:**
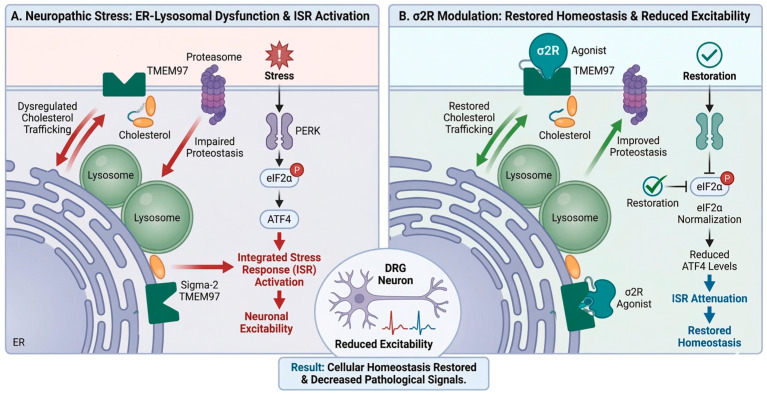
σ2R involvement in neuropathic pain.

In neuropathic pain, chronic ISR activation promotes ATF4-dependent transcriptional remodeling that increases excitability and reduces metabolic resilience [109]. The accumulation of metabolic stress in DRG neurons directly links these upstream lipids and proteostatic disturbances to nociceptive hypersensitivity. σ2R/TMEM97 modulators reduce p-eIF2α, attenuate ATF4 signaling, and restore proteostatic balance [99] (Figure 6). For instance, highly selective σ2R ligands, such as FEM-1689 and UKH-1114, have demonstrated robust anti-allodynic effects by specifically inhibiting the ISR in DRG neurons in a σ2R/TMEM97-dependent manner [103,110]. These effects distinguish σ2R/TMEM97 ligands from conventional analgesics: rather than modulating excitability directly through classical ion channel blockade, they target upstream homeostatic disturbances—such as cholesterol trafficking bottlenecks and ER/lysosomal stress—that drive chronic pain [104] (Figure 6).

### 4.3. Sigma 2 Receptor Ligands

**Figure 7:**
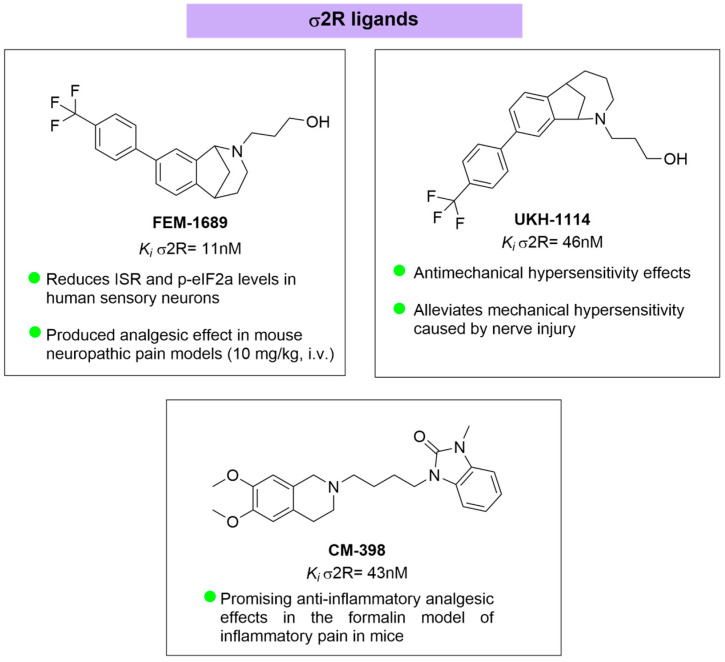
σ2R ligands.

- FEM-1689 is the most extensively characterized σ2R/TMEM97 ligand (K*_i_* = 11 nM) and provides definitive evidence of σ2R/TMEM97-dependent analgesia. Its efficacy is abolished in σ2R/TMEM97-knockout mice, establishing strict receptor dependence. FEM-1689 acts as a σ2R/TMEM97-dependent ISR suppressor, reducing p-eIF2α and ATF4 in DRG neurons and reversing SNI-induced mechanical allodynia [102].
- UKH-1114 (K*_i_* = 46 nM) displays potent anti-allodynic efficacy with an unusually long duration of action (>48 h), suggesting engagement of slow metabolic or transcriptional pathways [103]. It functions as a homeostatic σ2R/TMEM97 modulator, not as an agonist or antagonist, and exemplifies the capacity of σ2R/TMEM97 modulation to induce durable analgesia.
- CM-398 (K*_i_* = 43 nM) exhibits strong CNS penetration and robust analgesic activity in multiple neuropathic models (CCI, SNI), without sedation or motor impairment [111]. Although modestly σ1R-active, its functional profile is dominated by σ2R/TMEM97-mediated modulation of metabolic and proteostatic pathways, making it a leading translational candidate (Figure 7).

### 4.4. Clinical Translation of σ2R Modulation

**Figure 8:**
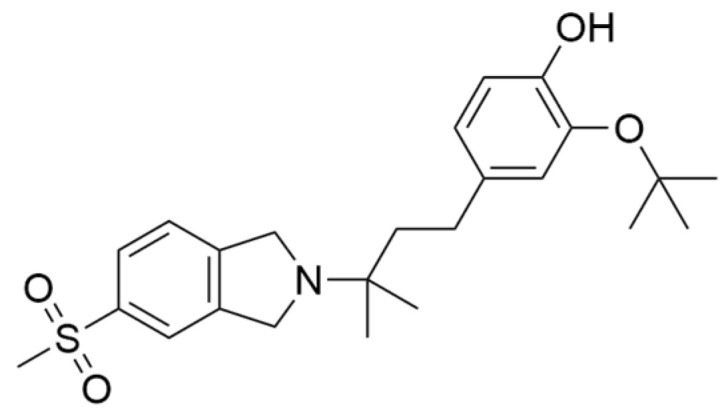
σ2R ligand CT1812.

Although no σ2R/TMEM97-selective compounds have entered clinical trials for neuropathic pain, the σ2R/TMEM97-binding drug CT1812, which remains in active Phase II clinical development for Alzheimer’s disease, demonstrates the feasibility and safety of σ2R/TMEM97 targeted modulation in humans [112,113] (Figure 8).

## 5. Comparative Integration of σ1R and σ2R in Neuropathic Pain

### 5.1. Biological Functions and Downstream Pathways

Despite being genetically unrelated and structurally divergent, σ1R and σ2R demonstrate a remarkable functional convergence, acting as complementary regulators of cellular resilience [114]. However, their downstream pathways are distinct. σ1R acts primarily as a ligand-operated molecular chaperone. Under stress conditions, it dissociates from the chaperone BiP/GRP78 and interacts with proteins like IP3R3 to regulate ER-mitochondria calcium transfer, mitigate oxidative stress (ROS clearance), and modulate the unfolded protein response (UPR) pathways [115,116,117]. In contrast, σ2R/TMEM97 plays a critical role in sterol homeostasis and acts as an external gateway for lipid uptake by forming a ternary complex with PGRMC1 and the low-density lipoprotein (LDL) receptor [108,118]. Together, they form a functional “Sigma Lipid Hub” where it mediates the external supply and cellular uptake of cholesterol, while σ1R manages its structural organization (e.g., stabilizing lipid rafts) and metabolic utilization within the cell.

### 5.2. Role in Neuropathic Pain

In the context of neuropathic pain, σ1R and σ2R do not function redundantly, nor do they act independently; rather, they exhibit a reciprocal and synergistic relationship [119]. Pharmacological and genetic studies reveal that these receptors play opposite roles in pain perception and opioid modulation. σ1R generally facilitates the development of neuropathic pain and acts as an endogenous inhibitor of MOR-mediated analgesia, which is why σ1R antagonists are highly effective antineuropathic agents. In contrast, σ2R promotes and facilitates MOR-mediated analgesia. Furthermore, σ2R agonists have emerged as robust antineuropathic agents, providing long-lasting relief from mechanical hypersensitivity in nerve injury models. Because they exchange regulatory roles during neuropathic pain, a combination of σ1R antagonism and σ2R agonism represents a powerful synergistic strategy for managing chronic pain states [110,119].

## 6. Therapeutic Outlook

Overall, the evidence reviewed here highlights σRs as therapeutic targets acting at distinct yet converging levels of neuropathic pain pathophysiology. Although σ1R and σ2R differ substantially in molecular identity and signaling logic, their pharmacological modulation converges on the normalization of maladaptive processes that sustain chronic pain states. σ1R antagonists primarily act by limiting pathological amplification of nociceptive signaling through the attenuation of ion channel sensitization, NMDA receptor–dependent synaptic plasticity, and neuroimmune activation within peripheral and central pain circuits. In parallel, σ2R/TMEM97 ligands operate upstream by restoring cellular homeostasis, dampening persistent activation of the ISR, and re-establishing proteostatic and metabolic balance in sensory neurons. Importantly, both receptor systems appear to selectively target pathological pain mechanisms while largely sparing physiological nociception, a feature that may translate into improved safety and tolerability profiles in comparison to conventional analgesics. While σ1R antagonists have already reached clinical evaluation in neuropathic pain and chemotherapy-induced neuropathy, σ2R/TMEM97 modulation remains at a preclinical stage; nevertheless, the clinical development of σ2R/TMEM97 ligands in other neurological disorders supports the translational feasibility of this approach. Together, these findings suggest that σRs–based therapies may offer a next-generation strategy to move beyond symptomatic pain relief toward disease-modifying modulation of neuropathic pain.

A critical, yet historically overlooked, dimension in the translational progress of σRs therapeutics is the consideration of Sex as a Biological Variable (SABV). While the possible role of sex-specific σ1R activation in the renoprotection against severe ischemia/reperfusion [120] and the significantly more potent effects in males of σ1 agonists in cardiac dysfunction induced by obstructive nephropathy [121] were investigated, the vast majority of foundational preclinical efficacy data for both σ1R and σ2R ligands in neuropathic pain indeed have been generated using almost exclusively male rodent models. Given the well-documented sexual dimorphism in chronic pain processing—including distinct neuroimmune interactions and differential reliance on microglial versus T-cell signaling pathways in the dorsal horn—this reliance on male subjects represents a significant gap in the current literature. However, emerging evidence underscores the importance of addressing SABV. For instance, recent investigations have highlighted sex-specific phenotypes, demonstrating that the loss of σ2R/TMEM97 exacerbates neuropathic injury-induced depression-like behaviors specifically in female mice [104]. Moving forward, it is imperative that future preclinical evaluations and clinical trial designs explicitly incorporate SABV. Determining whether σ1R and σ2R modulators exhibit sex-dependent pharmacological efficaciacy or pharmacokinetic differences will be essential to ensure that these next-generation targeted therapies are safe and effective across diverse patient populations.

## Figures and Tables

**Table 1:** Anatomical and cellular distribution of σ1R in pain circuits.

*Anatomical* *Region/Circuit*	Cellular Localization of σ1R	Functional Contribution to the Pain Process	Implications for Targeted σ1R Modulation	Species (Sex)
*Dorsal root ganglia (DRG)*	Small–medium primary sensory neurons; satellite glial cells	Stress-induced pro-excitatory chaperone activity enhances nociceptor excitability and peripheral neuroinflammation	σ1R antagonists reduce ion-channel trafficking, dampen inflammation, and prevent peripheral sensitization.	Rat (Male/ Female)
*Peripheral nerve/Schwann cells*	Myelinating and non-myelinating Schwann cells	Injury-induced glial activation sustaining a pro-nociceptive peri- axonal environment	σ1R blockade limits Schwann cell reactivity and ectopic nociceptive input	Rat (Male/ Female)
*Spinal dorsal horn (laminae I–II)*	Postsynaptic neurons; activated astrocytes and microglia	Facilitation of central sensitization via enhanced excitation and reduced inhibition	σ1R antagonists suppress wind-up and restore excitatory/inhibitory balance	Rat (Male)
*PAG/RVM*	Descending modulatory neurons	Negative modulation of endogenous opioid- mediated analgesia	σ1R antagonism enhances descending analgesia and opioid efficacy	Rat (Male)
*Anterior cingulate cortex (ACC)*	Pyramidal neurons; cortical microglia	Regulation of pain unpleasantness and affective amplification	σ1R modulation attenuates affective and cognitive pain comorbidities	Mouse (Unspecified)
*Amygdala*	Basolateral and central nuclei	Promotion of pain-related fear and aversive memory	σ1R antagonists reduce anxiety and fear responses linked to pain	Mouse (Unspecified)
*Nucleus accumbens (NAc)*	Medium spiny neurons; dopaminergic terminals	Dysregulation of reward and motivation in chronic pain	σ1R antagonism restores motivational and affective balance	Mouse (Female)

**Table 2:** Summary of Classical and Analytical σ1R Antagonists for Neuropathic Pain.

Ligand	Structural Class	Receptor Affinity (K*_i_*)	Selectivity Profile	Key Behavioral Outcomes in NP Models
**Haloperidol**	Butyrophenone	σ1R = 2–6 nM	Non-selective; high affinity for dopamine D2 receptors	Reduces mechanical allodynia/hyperalgesia in CCI and STZ-DPN models; limited by extrapyramidal side effects
**BD 1047**	Phenylaminoethyl-piperazine	σ1R = 0.9 nM	Highly selective over dopaminergic and opioid receptors	Reduces allodynia in bone cancer pain, SNL, and CCD models; inhibits spinal microglial activation
**LMH-2**	Haloperidol analog	σ1R = 6.0 nM	58-fold lower affinity for D2 compared to HAL; potential TRPV1 interaction	Antiallodynic/. antihyperalgesic efficacy superior to gabapentin in NA-STZ mice without motor side effects
**(+)-MR200**	Cyclopropyl- methyl-piperidine	σ1R = 1.5–4.0 nM	Highly selective; lacks dopaminergic affinity	Reverses allodynia and hyperalgesia in CCI and carrageenan models; normalizes Connexin 43 expression
**E-52862 (S1RA)**	1-Arylpyrazole	σ1R = 17 nM	Exceptional selectivity (σ1R/σ2R > 550); negligible affinity for 170+ targets	Inhibits spinal wind-up and pro-inflammatory cytokine expression; effective in OIN, STZ-DPN, and nerve injury models

**Table 3:** Anatomical and cellular distribution of σ2R/TMEM97 in pain circuits.

*Anatomical Region/Circuit*	Cellular Localization of TMEM97	Functional Contribution to Pain Processing	Implications for σ2R Targeted Modulation	Species (Sex)
*Dorsal Root Ganglia (DRG)*	Small- and medium-diameter nociceptors (C- and Aδ-fibers); satellite glial cells	High vulnerability to metabolic and proteostatic stress; initiation of peripheral sensitization	σ_2_R ligands reduce ISR hyperactivation and restore neuronal homeostasis, lowering nociceptor excitability	Mouse, Human (Male, Female)
*Peripheral Nerve/Schwann Cells*	Myelinating and non- myelinating Schwann cells	Axonal metabolism, lipid regulation, and trophic support; involvement in neuropathic nerve remodeling	σ_2_R modulators stabilize glial–axonal interactions and reduce stress-induced Schwann cell dysfunction	Mouse (Male, Female)
*Spinal Dorsal Horn (Laminae I–II)*	Presynaptic nociceptor terminals; interneurons (including inhibitory populations)	Synaptic integration of nociceptive input; central sensitization and disinhibition	σ_2_R activation restores proteostatic balance, dampens synaptic hyperexcitability, and supports inhibitory circuit function	Mouse, Human (Male, Female)
*Thalamus*	Relay nuclei involved in sensory-discriminative pain processing	Transmission and amplification of nociceptive signals to the cortex	σ_2_R modulation normalizes thalamic stress responses and reduces sensory amplification	Mouse (Unspecified)
*Periaqueductal Gray (PAG)*	Neurons in descending modulatory pathways	Coordination of endogenous analgesic responses; descending facilitation under chronic pain	σ_2_R ligands may rebalance descending modulation by reducing ISR-driven maladaptation	Mouse (Male, Female)
*Anterior Cingulate Cortex (ACC)*	Excitatory neurons; limbic integrative circuits	Affective-motivational pain components; cognitive modulation of nociception	σ_2_R modulation reduces stress-related affective amplification and maladaptive emotional weighting of pain	Mouse (Unspecified)
*Amygdala*	Basolateral and central nuclei	Pain-related fear, anxiety, aversion, and emotional memory	σ_2_R ligands counteract ISR-driven affective sensitization and stress-related hypervigilance	Mouse (Male, Female)
*Insular Cortex*	Granular and dysgranular regions	Interoception, pain unpleasantness, and integration of sensory and affective signals	σ_2_R modulation may attenuate chronic pain– induced dysregulation of interoceptive salience	Mouse (Male, Female)

## Data Availability

Dataset available on request from the authors.
